# Dissecting the Genetic Architecture of Aphanomyces Root Rot Resistance in Lentil by QTL Mapping and Genome-Wide Association Study

**DOI:** 10.3390/ijms21062129

**Published:** 2020-03-20

**Authors:** Yu Ma, Afef Marzougui, Clarice J. Coyne, Sindhuja Sankaran, Dorrie Main, Lyndon D. Porter, Deus Mugabe, Jamin A. Smitchger, Chongyuan Zhang, Md. Nurul Amin, Naser Rasheed, Stephen P. Ficklin, Rebecca J. McGee

**Affiliations:** 1 Department of Horticulture, Washington State University, Pullman, WA 99164, USA; yu.ma@wsu.edu (Y.M.); dorrie@wsu.edu (D.M.); stephen.ficklin@wsu.edu (S.P.F.); 2 Department of Biological Systems Engineering, Washington State University, Pullman, WA 99164, USA; afef.marzougui@wsu.edu (A.M.); sindhuja.sankaran@wsu.edu (S.S.); chongyuan.zhang@wsu.edu (C.Z.); 3 USDA-ARS Plant Germplasm Introduction and Testing Unit, Washington State University, Pullman, WA 99164, USA; clarice.coyne@usda.gov; 4 USDA-ARS Grain Legume Genetics and Physiology Research Unit, Prosser, WA 99350, USA; lyndon.porter@usda.gov; 5 Department of Crop and Soil Sciences, Washington State University, Pullman, WA 99164, USA; deus.mugabe@wsu.edu (D.M.); jsmitchger@wsu.edu (J.A.S.); 6 Breeder Seed Production Center, Bangladesh Agricultural Research Institute, Debiganj-5020, Panchagarh, Bangladesh; mdnurul.amin@wsu.edu; 7 Institute of Soil and Environmental Sciences, University of Agriculture, Faisalabad 38000, Pakistan; nasirrasheed@ymail.com; 8 USDA-ARS Grain Legume Genetics and Physiology Research Unit, Pullman, WA 99164, USA

**Keywords:** *Aphanomyces euteiches*, candidate genes, GBS, GWAS, lentil, linkage disequilibrium (LD), QTL mapping, root rot, SNP

## Abstract

Lentil (*Lens culinaris* Medikus) is an important source of protein for people in developing countries. Aphanomyces root rot (ARR) has emerged as one of the most devastating diseases affecting lentil production. In this study, we applied two complementary quantitative trait loci (QTL) analysis approaches to unravel the genetic architecture underlying this complex trait. A recombinant inbred line (RIL) population and an association mapping population were genotyped using genotyping by sequencing (GBS) to discover novel single nucleotide polymorphisms (SNPs). QTL mapping identified 19 QTL associated with ARR resistance, while association mapping detected 38 QTL and highlighted accumulation of favorable haplotypes in most of the resistant accessions. Seven QTL clusters were discovered on six chromosomes, and 15 putative genes were identified within the QTL clusters. To validate QTL mapping and genome-wide association study (GWAS) results, expression analysis of five selected genes was conducted on partially resistant and susceptible accessions. Three of the genes were differentially expressed at early stages of infection, two of which may be associated with ARR resistance. Our findings provide valuable insight into the genetic control of ARR, and genetic and genomic resources developed here can be used to accelerate development of lentil cultivars with high levels of partial resistance to ARR.

## 1. Introduction

Lentil (*Lens culinaris* Medikus), an important grain legume, is widely grown throughout the world with an annual production of 7.8 million tons [1]. In 2017, the major producers of lentils were Canada (48.1%), India (15.7%), Turkey (5.5%), United States (4.4%), and Kazakhstan (4.0%). Lentils have high concentrations of protein, minerals, carbohydrates, and fiber and are an inexpensive food that can help alleviate malnutrition in developing countries. Through a symbiotic association with *Rhizobium leguminosarum*, lentils are able to fix atmospheric nitrogen, which offers significant benefits in cereal-based cropping systems [2].

Root rot disease caused by *Aphanomyces euteiches* Drechs. is one of most devastating diseases in lentil production and can cause yield losses up to 80% [3]. *A. euteiches* belongs to the phylum Oomycota, and this soil-borne pathogen has a wide host range within Fabaceae, including pea, lentil, faba bean, dry bean, alfalfa, and red clover [3,4,5,6]. Species-specific *A. euteiches* isolates were reported by Moussart et al. [7], Malvick and Percich [8], and Levenfors et al. [9]. Aphanomyces root rot (ARR) was first described by Jones and Drechsler in 1927 and was reported as a prevalent pathogen in pea fields worldwide. Kraft et al. [10] determined that the progress of the disease in lentil was rapid and severe, and none of the cultivars and the accessions they evaluated were resistant. Further testing of lentil cultivars with a French isolate confirmed the lack of resistance in lentil lines [7]. While it was known previously, lentil root rot caused by *A. euteiches* was first identified in the US in 2008 [11] and in Canada in 2012 [12]. It is now recognized to be widespread in lentil growing areas across most of the northern Great Plains of the U.S. and Canada [13,14]. The severe root damage caused by *A. euteiches* dramatically restricts water and nutrient transport from the roots and results in stunted plants and tremendous yield loss [15]. The thick-walled oospores can survive in soil more than 10 years [16], making crop rotation an ineffective method of disease management. Given that limited information exists regarding the pathogenic variability and the physiologic specialization of this pathogen, comprehensive races of *A. euteiches* have not been identified [17]. Nevertheless, differences in the ability of different isolates to infect plants were well characterized, and two Pathotypes (I and III) were identified. Pathotypes I and III were found in North America, while only Pathotype I was documented in France [18]. Cultural practices, fungicides, biological control, and soil fumigants are undesirable approaches to manage ARR, as they were proven to be either ineffective or environmentally unfriendly [15]. The most effective, economical, and sustainable management of ARR is utilizing genetic resistance in the development of cultivars with high levels of partial resistance. 

However, no lentil cultivars resistant to ARR are currently available, which makes economic loss and the costs of managing this disease significant. Developing other legume cultivars with resistance to ARR through traditional breeding has been hampered by the difficulty of pyramiding resistance genes given the polygenic nature of inheritance [19]. A genome-wide approach provides an unprecedented opportunity for breeding programs to accelerate the development of lentil cultivars with high levels of resistance to ARR. Limited genetic and genomic resources have been available for lentil due to its relatively large genome (~4.2 GB) and minor crop status. Recently, an international collaboration developed a draft lentil genome reference [20] and the advent of inexpensive, high-throughput sequencing and marker development made possible genetic and genomic research required to investigate highly quantitative characteristics in lentil [21]. Quantitative trait loci (QTL) analysis, by integrating genome-wide association study (GWAS) and QTL mapping, is an efficient and effective approach to unravel genetic architecture and detect variations underlying complex traits, particularly polygenic resistance to plant disease [22,23,24]. Over the last two decades, QTL mapping has become a powerful tool in identifying genomic regions associated with traits of interests in bi-parental populations. The low allelic diversity and recombination rates in bi-parental populations, however, limit the ability to detect natural variations in diverse genetic backgrounds. To overcome the limitations inherent in QTL mapping, GWAS is able to assess wider genetic diversity and probe greater amounts of recombination due to the evolutionary history of natural populations. By mitigating each other’s limitations, combining the two QTL analysis approaches provides a complementary, powerful, and robust assay to uncover the genetic basis underlying complex traits [25]. Moreover, with the advent of plant sensing technologies, image-based high-throughput phenotyping enables acquisition of high-quality phenotypic data rapidly, accurately, and objectively [26]. Integration of traditional and image-based phenotyping tools offers us a robust assay to uncover the genetic basis underlying complex traits.

Due to the relatively recent identification of ARR and the lack of genomic resources in lentil, no QTL studies on this disease have been reported in lentil to date. Conversely, the genomic regions associated with ARR resistance in pea and *Medicago* are well characterized. Twenty-seven meta-QTL were identified in pea in four recombinant inbred line (RIL) populations, seven of which were major resistance QTL detected consistently across multiple environments [27]. Subsequently, GWAS validated seven major resistance QTL and discovered numerous novel QTL associated with resistance to ARR in pea [19,28]. In *Medicago truncatula*, a candidate gene encoding an F-box protein was identified, using GWAS, as a negative regulator of resistance to ARR [29]. To bridge the gap between ARR genetic and genomic knowledge and breeding application in lentil, in the present work, we performed QTL mapping and GWAS to discover genomic regions associated with genetic resistance to *A. euteiches* in lentil. We investigated the genetic variations in a USDA lentil single plant-derived (LSP) core collection, an international collection from the International Center for Agricultural Research in the Dry Areas (ICARDA), and an RIL population derived from a susceptible line and a partially resistant line. We integrated large-scale single nucleotide polymorphism (SNP) data generated via genotyping by sequencing (GBS) and high-throughput image-based phenotyping to decipher the genetic architecture. This study provides promising insights of ARR resistance in lentil and will accelerate the development of resistant cultivars. 

## 2. Results

### 2.1. Phenotypic Data Analysis

**Figure 1:**
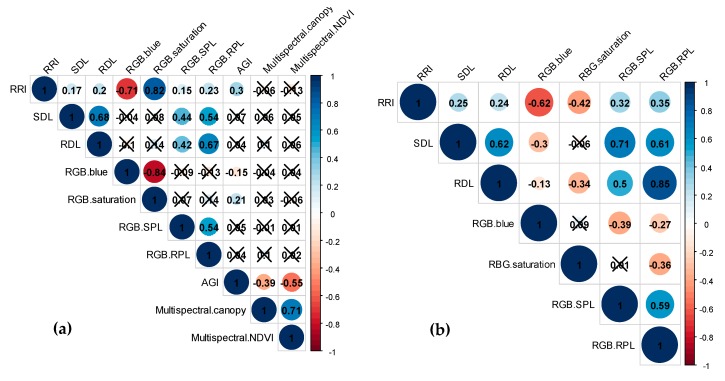
Heatmaps of correlation coefficients between the ARR resistance traits in (**a**) the RIL population and (**b**) the association mapping population. Blue color represents positive correlation and red color indicates negative correlation. Cross mark shows there is no significant correlation observed between the given traits (*p* > 0.05).

The RIL population evaluated for reaction to *A. euteiches* in a controlled condition (189 RILs) and an infested field condition (173 RILs) displayed transgressive segregation for all the resistance traits (Supplementary Figure S1). The analysis of variance (ANOVA), shown in Table 1, indicated all the genotypes had significant differences for each of the resistance traits (*p* < 0.05). In terms of the environmental (replicate) effects, there were no significant differences for most of the traits; however, shoot dry weight loss per plant (SDL), root dry weight loss per plant (RDL), average intensity of blue channel acquired from an Red-Green-Blue (RGB) camera (RGB.blue), above ground index (AGI), and canopy area acquired from a multispectral camera (Multispectral.canopy) showed significant environmental effects. Broad-sense heritability appeared to be relatively low in the RIL population and ranged from 0.05 (Multispectral.canopy) to 0.52 (RGB.blue) (Table 1). Frequency distributions of most traits fit normal curves, whereas SDL, RDL, number of pixels loss per plant in shoot acquired from an RGB camera (RGB.SPL), and number of pixels loss per plant in root acquired from an RGB camera (RGB.RPL) had moderately skewed distributions toward high scores. All the traditional ARR traits, root rot index (RRI), SDL, RDL, and AGI, were significantly and positively correlated with each other (*p* < 0.01) except between AGI and SDL/RDL. The correlation coefficient analysis showed correlations between the two environments were relatively low (0.15 <|*r*|< 0.30). Image-derived traits, RGB.blue, standard deviation of saturation channel acquired from an RGB camera (RGB.saturation), RGB.SPL, RGB.RPL, standard deviation of normalized difference vegetation index acquired from a multispectral camera (Multispectral.NDVI), and Multispectral.canopy, were significantly and highly correlated with corresponding traditional ARR traits (0.15 < |*r*| < 0.82) (Figure 1).

Using a GWAS approach, 326 diverse accessions were evaluated for *Aphanomyces* resistance under controlled conditions at the seedling stage. ANOVA of all the traits indicated highly significant genotype effects (*p* < 0.001) and environmental (replicate) effects (*p* < 0.001) (Table 1). Frequency distributions of SDL and RGB.RPL fit a normal curve, while RRI, RGB.saturation, and RGB.SPL had moderately skewed distributions toward low scores, and RDL and RGB.blue skewed toward high scores (Supplementary Figure S1). Broad-sense heritability was high for RRI and RGB.saturation (*H^2^* = 0.73) but moderate for the rest of the traits (0.50 < *H^2^* < 0.62). All the traditional ARR traits (RRI, SDL, and RDL) of the association mapping population were significantly and positively correlated with each other (*p* < 0.001), where the correlation coefficient was higher between SDL and RDL (*r* = 0.62) than between RRI and SDL (*r* = 0.25) and RRI and RDL (*r* = 0.24). Image-derived traits (RGB.blue, RGB.saturation, RGB.SPL, and RGB.RPL) were significantly and highly related with traditional ARR traits with |*r*| ranging from 0.13 to 0.85, especially between RDL and RGB.RPL (*r* = 0.85) (Figure 1).

### 2.2. Genotypic Data Analysis 

GBS libraries of the RIL population were constructed using the two-enzyme GBS approach and sequenced on an Illumina HiSeq^TM^ 4000 platform. A total of 3.9 billion reads were obtained from the high-throughput sequencing (Supplementary Table S1), and 2.9 billion reads (74% of raw data) were retained after demultiplexing and cleaning. The average number of reads per sample was 15 million with a range from 5.1 million to 34 million reads. With Freebayes-based SNP calling, 164,099 SNPs were identified among the two parents and the RILs. Those SNPs that were polymorphic between the two parents without unknown and heterozygous genotypes, with minor allele frequency (MAF) > 0.3, and with missing values < 0.2 were retained, leaving 2880 SNPs for analysis. 

For the association mapping population, the same two-enzyme approach was used for GBS library construction, but the libraries were sequenced on an Illumina HiSeq^TM^ 2500 platform. Subsequently, 1.7 billion raw reads were generated through the sequencing platform (Supplementary Table S1), and 1.5 billion cleared reads (86% of raw data) were retained. The number of reads per sample ranged from 1.4 million to 11.6 million with an average of 4.5 million reads. Using the Stacks pipeline, 28,593 SNPs were identified in this population. With filtering the SNPs with MAF > 0.05 and missing values < 0.3, 4558 SNPs were retained for further GWAS.

### 2.3. QTL Mapping

In total, 2865 out of 2880 SNPs identified from the RIL population were assigned to seven linkage groups. The estimated map had a length of 978.1 cM and a density of 2.9 markers per cM with 99% of the intervals between adjacent markers being smaller than 5 cM (Supplementary Table S2). QTL associated with ARR were identified across all the chromosomes except Chr 1. A total of two QTL were identified for RRI, one QTL for SDL, and two QTL for AGI. In terms of image-derived features, three QTL for RGB.blue, three QTL for RGB.saturation, one QTL for RGB.SPL, two QTL for RGB.RPL, two QTL for Multispectral.canopy and three QTL for Multispectral.NDVI were identified (Table 2). The detected QTL explained from 5.2% to 12.1% of the phenotypic variance. It is worthwhile to note that two co-localization regions were identified on Chr 2 and Chr 5, respectively, where five QTL (Q.RRI-Lc2.1, Q.BLU-Lc2.1, Q.SAT-Lc2.1, Q.CAN-Lc2.1, and Q.AGI-Lc2.1) were clustered between 22.8 cM and 31 cM on Chr 2 and two QTL (Q.RRI-Lc5.1 and Q.AGI-Lc5.1) were co-localized between 51.7 cM and 64.8 cM on Chr 5.

### 2.4. Genome-Wide Association Study

**Figure 2:**
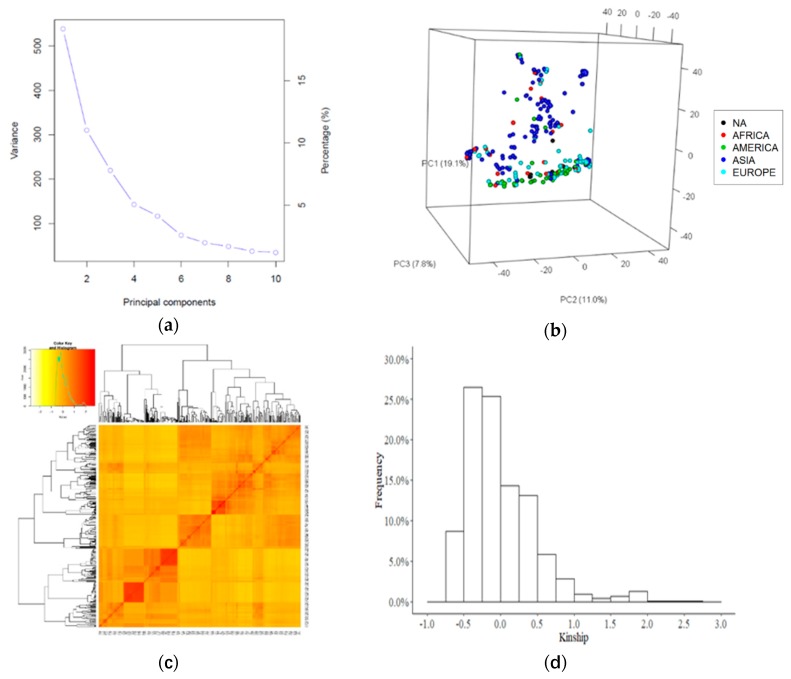
Population structure of the association mapping population. (**a**) The scree plot of first 10 principle components (PCs). (**b**) The three-dimensional principle component analysis (PCA) plot represents the distribution of the lentil accessions on the first three PCs. The colors represent various continents: red-Africa, green-America, blue-Asia, light blue-Europe, and black-data not available. (**c**) Cluster heatmap of the Kinship matrix. (**d**) Histogram of kinship coefficients.

With an analysis using unimputed 4558 SNPs in Haploview, linkage disequilibrium (LD) decay was estimated to range from 128 kb to 762 kb with an average of 331 kb over all the chromosomes (Supplementary Figure S2). In the principle component (PC) analysis, the first three PCs explained 19.1%, 11%, and 7.8% (37.9% total) of genetic variance (Figure 2a,b) and clearly separated the lentil accessions collected in Asia from other regions (Figure 2b). The first three PCs were used as covariates to account for population structure in GWAS. The VanRaden kinship coefficient matrix (Figure 2c) ranged from −0.68 to 2.78, with 79% of pairwise kinship coefficients ranging from −0.5 to 0.5 (Figure 2d).

GWAS identified a total of 38 QTL across seven chromosomes associated with five ARR related traits, including two traditional phenotypic traits (RRI and RDL) and three image-derived traits (RGB.saturation, RGB.blue, and RGB.RPL). The *p*-values ranged from 3.4 × 10^−10^ to 8.9 × 10^−4^, explaining 1.4% to 21.4% of phenotypic variance (Table 3). Nine QTL exceeded the experiment-wise threshold *p* < 2.2 × 10^−6^, 13 reached the marker-wise threshold *p* < 0.0001, and 16 had a marker-wise *p*-value between 0.0001 and 0.001. Four co-localization regions were detected on four chromosomes. Two QTL (G.RRI-Lc1.1 and G.BLU-Lc1.1) were co-localized on Chr 1 with a highly significant trait-associated SNP marker (1569_6) consistently detected for RRI and RGB.blue, explaining 10.7% and 13.7% of the phenotypic variance, respectively. A co-localization region on Chr 4 included two QTL (G.RDL-Lc4.1 and G.RPL-Lc4.2) for RDL and RGB.RPL. Two QTL (G.RRI-Lc5.1 and G.SAT-Lc5.1) were clustered on Chr 5 where a trait-associated SNP marker (7154_47) was consistently detected for RRI and RGB.saturation, explaining 10% of phenotypic variance for RRI. A co-localization region was identified on Chr 6 for RDL, RGB.saturation, and RGB.RPL (Table 3).

### 2.5. LD Block Haplotypes

**Figure 3:**
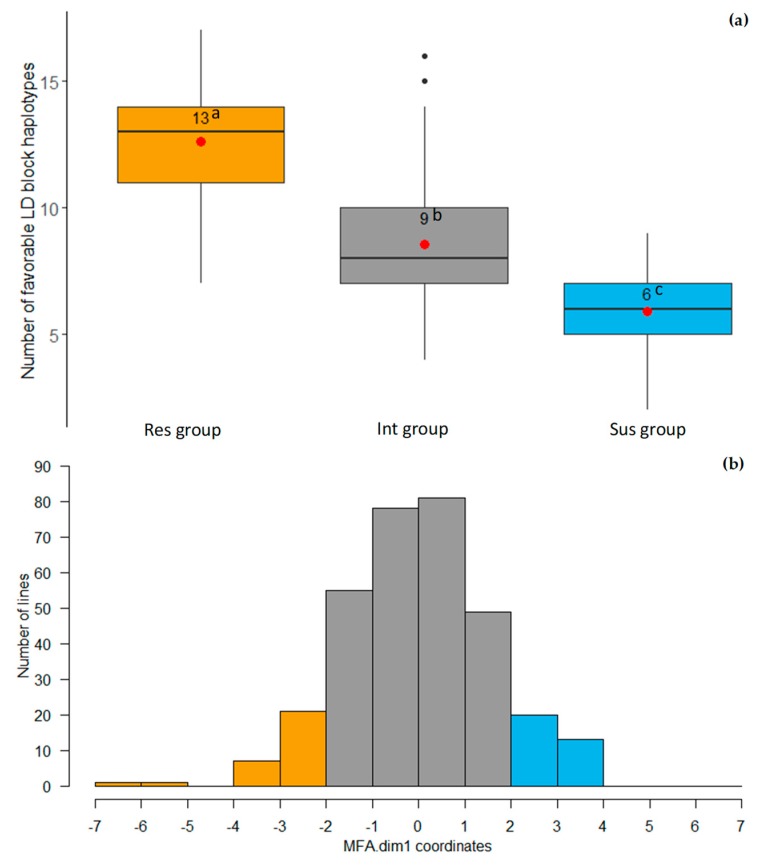
Comparison of numbers of favorable LD block haplotypes in resistant (Res), intermediate (Int), and susceptible (Sus) groups in the association mapping population. (**a**) A box plot of the number of favorable LD block haplotypes among three groups. The three groups were categorized by coordinates of first PC of multiple factor analysis (MFA.dim1). Numbers in the box plot indicate mean values of numbers of favorable haplotypes in the given groups. Letters represent significant differences among the three groups based on the mean comparison. (**b**) Frequency distribution of MFA.dim1 in the association mapping population. Res group (MFA.dim1 < −2, yellow color) carried the highest number of favorable haplotypes, Int group (−2 < MFA.dim1 < 2, gray color) had an intermediate number of favorable haplotypes, and Sus group (MFA.dim1 > 2, blue color) carried the lowest number of favorable haplotypes.

Thirty-three LD blocks were identified containing all the markers in LD *r^2^* > 0.5 with marker-trait associations (MTAs). Three to 13 haplotypes were detected within each of the LD blocks. Twenty-seven favorable haplotypes and 30 unfavorable haplotypes were selected in accordance with comparison of phenotypic mean values between each group (Supplementary Table S3). Among the association mapping population, the ARR resistant lentil lines with multiple factor analysis coordinates (MFA.dim1) < −2 (13 favorable haplotypes on average) had a significantly higher number of favorable haplotypes than the intermediate lines (nine favorable haplotypes on average) and the susceptible lines (six favorable haplotypes on average) (Figure 3). Lentil lines ILL_1861 and ILL_4830 carried the 17 favorable LD block haplotypes, whereas PI_432237 and PI_299215 had only two favorable haplotypes (Supplementary Table S4). 

### 2.6. QTL Clusters

**Figure 4:**
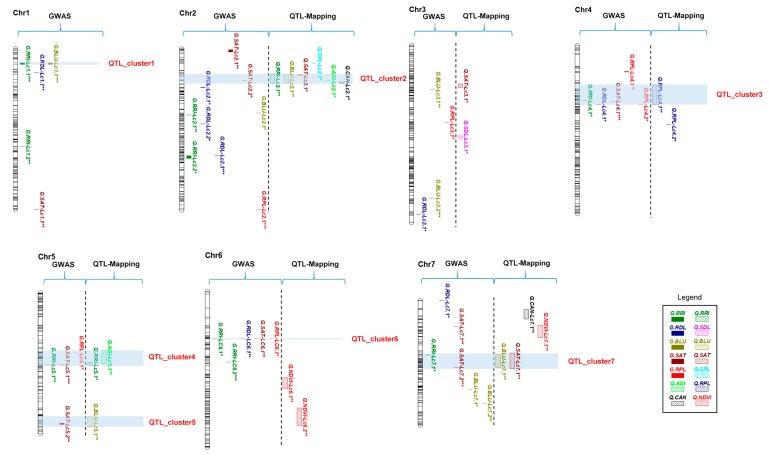
Genetic map of ARR resistance QTL identified using QTL mapping and genome-wide association study (GWAS). The solid bars represent GWAS-based QTL, and the hollow bars indicate QTL-mapping-based QTL. QTL clusters are highlighted by blue-shaded banding. QTL significance levels are labeled using *** for major QTL, ** for nominal QTL, * for suggestive QTL.

According to the GWAS and the QTL mapping results, seven clusters (QTL_cluster1- QTL_cluster7) were defined on Chr 1, Chr 2, Chr 4, Chr 5, Chr 6, and Chr 7 (Figure 4), where the cluster was considered as a co-localization region consisting of at least two QTL identified using GWAS and/or QTL mapping. Among the seven clusters, five contained QTL from both GWAS and QTL mapping, whereas two (QTL_cluser1 and QTL_cluster6) were only GWAS-based clusters. Furthermore, QTL_cluster2, QTL_cluster3, QTL_cluster4, and QTL_cluster7 were major QTL clusters. QTL_cluster2 included six QTL for five traits, including two traditional phenotypic traits (RRI and AGI) and three image-derived traits (RGB.blue, RGB.saturation, and Multispectral.canopy), with a GWAS-based trait-associated SNP marker (3450_21) explaining 21% of phenotypic variance. QTL_cluster3 and QTL_cluster4, the second major clusters, each contained five QTL. QTL_cluster7 consisted of four QTL for three traits with a trait-associated marker explaining 21.4 % of phenotypic variance.

### 2.7. Prediction of Candidate Genes and Expression Analysis

With annotations of 106 SNPs identified in the 33 ARR-related LD blocks, 92 SNPs were located in genes, 43 of which were in coding sequences, 42 were in introns, and seven were in untranslated regions (Supplementary Table S5). In addition, 34 SNPs were discovered in the seven QTL clusters and assigned to 15 types of genes. Among these genes, five were chosen for validation, encoding Leucine Rich Repeat Receptor-like Kinase (LRR-RLK), cytochrome P450 family 71 protein (CYP71), ATP binding cassette transporter A family protein (ABCA), pectin esterase (PE), and chalcone-flavanone isomerase family protein (CHI). It was reported in previous studies that these genes are potentially associated with ARR resistance in pea [19] and *Medicago* [29,30]. 

**Figure 5:**
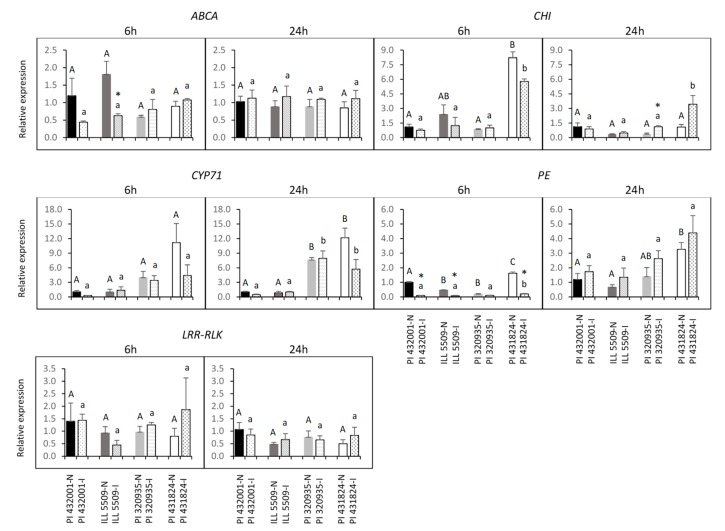
Expression profiles of putative genes. Relative expression levels were normalized by ß-tubulin-3. Four accessions (two partially resistant accessions: PI 432001 and ILL 5509; two susceptible accessions: PI 320935 and PI 431824) were analyzed in non-inoculated (N) and inoculated (I) with *A. euteiches* conditions at two time-points (6 and 24 hours post inoculation: 6 hpi and 24 hpi). Error bars represent standard error of the mean. Asterisks indicate statistically significant differences (*p* < 0.05) between inoculated plants and non-inoculated plants. Capital letters represent significant differences among all four accessions under the non-inoculated condition, while lowercase letters indicate significant differences among all four accessions under the inoculated condition. Abbreviations: *ABCA* gene encoding ABC transporter A family protein (ABCA), *CYP71* gene encoding Cytochrome P450 family 71 protein (CYP71), *LRR-RLK* gene encoding LRR receptor-like kinase (LRR-RLK), *CHI* gene encoding Chalcone-flavanone isomerase family protein (CHI), and *PE* gene encoding Pectin esterase (PE).

Expression profiles of five candidate genes were examined among four lentil accessions (partially resistant accessions: PI 432001 and ILL 5509; and susceptible accessions: PI 320935 and PI 431824) during interaction with *A. euteiches* at 6 hpi (hours post inoculation) and 24 hpi (Figure 5). Expression of *ABCA* was significantly (*p* < 0.05) downregulated three-fold in the resistant accession ILL 5509 at 6 hpi in response to *A. euteiches*, while there was no significant difference (*p* > 0.05) between inoculated and non-inoculated plants in the resistant accession PI 432001 at the same time point. No expression polymorphism was observed in the four accessions at 24 hpi. *CHI* expression level was significantly up-regulated (2.8-fold) in the susceptible accession PI 320935 at 24 hpi, but there was no significant change at 6 hpi. Surprisingly, expression of *CHI* was much higher in the susceptible accession PI 431824 than other accessions at 6 hpi under both inoculated and non-inoculated conditions. Expression of *CYP71* was higher in the susceptible accessions than resistant accessions at 24 hpi, but no significant expression difference was observed between inoculated and non-inoculated plants in any of the accessions. It indicated that such expression differences are ecotype-specific and may not relate with ARR disease. *PE* was more dramatically suppressed in the resistant accessions PI 432001 (10-fold) and ILL 5509 (five-fold) than the susceptible accession (two-fold) at 6 hpi, but the gene expression level was significantly downregulated (eight-fold) in the susceptible accession PI 431824. Expression of *LRR-RLK* was unchanged in the four accessions at both time points. The quantitative reverse transcriptase-polymerase chain reaction (qRT-PCR) analysis revealed that *ABCA*, *CHI,* and *PE* were differentially expressed after *A. euteiches* inoculation, and two, *ABCA* and *PE*, may be associated with ARR resistance. *CYP71* and *LRR-RLK* may not be directly linked to this disease.

## 3. Discussion

Genetic and genomic resources for lentil have been limited due to its relatively large genome size (~4.2 GB) [31]. However, with the emerging technologies in DNA sequencing, this is changing rapidly, including online access to the lentil reference genome for lentil cultivar CDC Redberry [20], an exome capture set for lentil [2], and assorted GBS data sets [32,33,34,35]. In this study, we demonstrated the first QTL mapping and GWAS in lentil to decipher the genetic basis of resistance to a major root disease, ARR, by evaluating two types of populations using advanced phenotyping and genotyping technologies. With the RIL population derived from two distinct parents and the association mapping population with a very wide genetic diversity, the two complementary QTL analysis approaches enabled us to precisely detect natural variations underlying this complex trait. We utilized GBS to discover novel SNPs for both populations, which significantly enhanced the genetic and the genomic resources available to help overcome the devastating impact of ARR in lentil production. Utilizing an advanced imaging-based phenotyping technology, we conducted the QTL analysis with image-derived features along with traditional phenotypic traits. Finally, our study reported novel QTL for resistance to ARR in lentil, pinpointed favorable haplotypes in the resistance lines, and identified putative candidate genes associated with ARR resistance. 

ARR is a complex polygenic trait with low heritability and is strongly affected by the environment [36,37]. Our study supports the observations of previous studies showing heritability ranged from 0.05 to 0.73 in the two mapping populations. With climate and soil structure as well as microbiome variations present in the field condition, the heritability shown in the field was lower than in the controlled conditions. In addition, a relatively low correlation was detected between these two conditions, as was also observed in the study of Pilet-Nayel et al. [38]. To effectively and precisely capture resistance variations among all the lentil lines and accessions, we integrated high-throughput phenotyping with RGB imaging and unmanned aerial system-based multispectral imaging in this study. With strong and significant correlations discovered between the image-derived traits and traditional phenotypic traits, the high-throughput phenotyping technology is considered an excellent alternative tool to phenotype this complex trait. 

QTL and association mapping approaches have proven to be complementary to and effective in detecting QTL across many species [39,40,41]. In the present study, we identified 19 QTL-mapping-based QTL and 38 GWAS-based QTL. Notably, limited co-localizations occurred among QTL discovered in the RIL population and the association mapping population. This highlighted the importance of integrating QTL mapping and association mapping for a comprehensive assessment of ARR resistance QTL. Seven QTL clusters were detected with at least two co-localized QTL found through GWAS and/or QTL mapping. Five clusters were both GWAS-based and QTL-mapping-based QTL. It is noteworthy that QTL_cluster2 on chromosome 2, QTL_cluster3 on chromosome 4, QTL_cluster4 on chromosome 5, and QTL_cluster7 on chromosome 7 accumulated the most QTL and explained large portions of the phenotypic variance. Interestingly, the majority of QTL within QTL_cluster2 were detected in the RIL population in both environments, but only one GWAS-based QTL for an image-derived trait (RGB.saturation) co-localized within this region. The saturation channel is in accordance with the pureness or the depth of certain colors that are difficult to detect with human eyes [42]. To some extent, therefore, the color features derived from imaging technology captured more information than the traditional traits, since RRI and AGI are subjective, and SDL and RDL are surrogate traits that may not be accurate when the disease symptoms have not been fully developed. 

With various molecular events underlying quantitative disease resistance, limited resistance QTL have been cloned, and resistance genes are largely unknown [43,44]. Diverse mechanisms underlying resistance QTL involve plant-pathogen recognition, signal transduction, plant development, and basal defense [44,45]. In our study, we explored five putative candidate genes selected from the QTL clusters using qRT-PCR. A putative candidate gene, *ABCA,* discovered in QTL_cluster7, was repressed in one of the resistant accessions at an early stage of infection. ABCA is a subfamily of ABC, one of the largest families of membrane proteins, and plays an important role in hormone transport, secondary metabolites, xenobiotics, and pathogen responses [46]. The functions of the plant ABCA protein subfamily are currently not well known. It was reported that *AtABCA1* functions in pollen germination and seed maturation germination in *Arabidopsis thaliana* [47,48] and *SlABCA1* are associated with secretion activity in tomato roots [49]. A transcriptome analysis of *M. truncatula* reported that genes encoding ABC transporters were significantly detected at one day post inoculation (dpi) with *A. euteiches* in both resistant and susceptible lines and repressed at 6 dpi only in the resistant line [30]. The findings in this *M. truncatula* study reflect the importance of *ABCA* as well, but the expression differences were observed at a later stage of infection. Further gene functional analysis, therefore, is essential to validate *ABCA* in response to *A. euteiches* in a future study.

We also identified a putative gene encoding PE in QTL_cluster7. Pectin is a critical component of plant cell walls, considered to be the first external barrier against pathogens [50]. PE is a cell wall degrading enzyme and has an important role in cell wall modification and breakdown by catalyzing pectin into pectate and methanol [51]. It was found that pectin methylesterase inhibitors enhance resistance to *Verticillium* wilt in cotton [52], *Fusarium graminearum* and *Bipolaris sorokiniana* in wheat [53], and *Botrytis cinerea* in *Arabidopsis* [54]. In addition, in a previous GWAS, *PE* was identified underlying a QTL associated with ARR resistance in pea [19]. In this study, we found that the expression of *PE* was more suppressed in the resistant accessions than one of the susceptible accessions (PI 320935) at 6 hpi but not in the other susceptible accession (PI 431824). Additional resistant and susceptible accessions, therefore, should be included in further gene expression studies to better clarify this conclusion. 

The other three genes, *CYP71, CHI,* and *LRR-RLK*, identified in QTL clusters may not be directly associated with the ARR resistance. For *CYP71*, expression differences were only elucidated between susceptible accessions and resistant accessions at 24 hpi but not between inoculated and non-inoculated plants in any of the accessions. This indicates that such gene expression differences could be ecotype-specific, and this gene may not play a direct role in response to *A. euteiches*. As for the second gene, *CHI* is a key gene in plant flavonoid biosynthesis, and flavonoids have been shown to be very important in plants’ resistance to pathogens [55,56]. Furthermore, in the transcriptome analysis of *M. truncatula*’s response to *A. euteiches*, flavonoid biosynthesis-related genes encoding chalcone synthase, chalcone O-methyltransferases, and isoflavonol reductase were significantly detected in resistant genotypes at 6 dpi [30]. In our study, however, we found *CHI* expression level was significantly upregulated in one of the susceptible accessions at 24 hpi. This result, inconsistent with previous studies, did not provide us enough assurance of *CHI*’s relatedness with ARR resistance. For the third gene, *LRR-RLK* expression was not changed in any of the accessions at either time point, indicating this gene may not be associated with ARR at an early stage of infection. 

## 4. Materials and Methods 

### 4.1. Plant Materials

An RIL population was developed from a cross between a breeding line (K192-1) with high partial resistance and a susceptible breeding line (K191-2). The two parents were selected from an ARR nursery in a farmer’s field near Kendrick, Idaho. The cross was made in Pullman, WA in 2013 and advanced using single-seed decent to the F_6_ generation. The final population contained 189 RILs and the two parents. 

The association mapping population consisted of 326 accessions from 60 countries on four continents, Asia (166), Europe (76), the Americas (47), Africa (30), and unknown origin (7). Among them, 109 accessions were from ICARDA, and 217 accessions were from the LSP collection obtained from the Western Regional Plant Introduction Station, USDA-ARS (Supplementary Table S6).

### 4.2. Inoculation Precedure

A pure culture of *A. euteiches*, isolate Ae-16-04D, belonging to Pathotype I (personal communication with Dr. Julie S. Pasche) was used in all controlled conditions. Prior to sowing, lentil seeds were surface sterilized in 95% ethanol for one minute followed by 10% bleach for one minute and then rinsed in distilled water. The seeds were individually planted in 8.25 cm deep Cone-tainers (Stuewe & Sons, Tangent, OR, USA) filled with perlite medium (AFCO Distribution & Milling, Spokane Valley, WA, USA). In order to produce the zoospores required for inoculation, the *A. euteiches* isolate was grown in pea broth and incubated in the dark at room temperature for six days. Mycelia mats were then removed from the pea broth, rinsed three times in distilled water, and incubated in mineral nutrient solution (0.26% CaCl_2_⋅2H_2_O, 0.49 MgSO_4_⋅7H_2_O, and 0.074% KCl) for 16 h to induce zoospore production [57]. The concentration of zoospores was calculated with a hemocytometer and adjusted to 10^4^ spores/mL. Fourteen-day old seedlings were inoculated with the zoospore suspension by pipetting 2 mL of inoculum at the base of each plant’s stem. The non-inoculated controls were “inoculated” with 2 mL of sterile distilled water.

### 4.3. Traditional and Image-Based Phenotyping under Controlled Condition

The RIL population and the association mapping population were evaluated in controlled conditions (25 °C 16 h/23 °C 8 h). The RIL population was planted using a completely randomized design with three inoculated replications and three non-inoculated replications (three plants/genotype/replication/treatment). The association mapping population was planted under the same controlled conditions in a randomized complete block design with 10 inoculated replications and 10 non-inoculated replications (one plant/genotype/replication/treatment). 

At 14 dpi with *A. euteiches*, plants were uprooted, and RRI was determined by assessing roots using a 0 (healthy plants) to 5 (dead plants) scoring scale adapted from McGee et al. [17]. Each entire plant was imaged using an RGB digital camera (Canon PowerShot SX530 HS, Canon U.S.A. Inc., Huntington, NY, USA), where one shoot feature (Shoot.projected.area-total number of pixels of shoots) and three root features (Root.projected.area-total number of pixels of roots, RGB.blue, and RGB.saturation) were extracted using MATLAB (MathWorks Inc., Natick, MA, USA), adopted from Marzougui et al. [58]. Shoots and roots were subsequently separated and placed in a forced-air drying room for seven days prior to collecting dry weights. As a surrogate measure of resistance, the dry weight loss of the inoculated vs. the non-inoculated plants was calculated. SDL and RDL were calculated with SDL/RDL = (dry weight per plant of non-inoculated treatment—dry weight per plant of inoculated treatment) / dry weight per plant of non-inoculated treatment. Subsequently, SDL and RDL were recorded using a 1 to 5 scoring scale, where 1 = greater than 100% of normal growth, 2 = 76–100% of normal growth, 3 = 51–75% of normal growth, 4 = 26–50% of normal growth, and 5 = 0–25% of normal growth [59]. In addition, RGB.SPL and RGB.RPL were calculated using the SDL/RDL formula described above and thereafter recorded via the same scale.

### 4.4. Traditional and Image-Based Phenotyping under Field Condition

The RIL population was assessed for *Aphanomyces* resistance in a naturally infested field near Kendrick, Idaho. The 173 RIL lines were planted using a randomized complete block design with three replications, 30 seeds/genotype/replicate. An adjacent susceptible control (Avondale) was planted every four lines and used to adjust for local disease variation using the formula as described in [60]. The plants were evaluated at flowering stage using AGI with a 1 (healthy plants) to 5 (dead plants) scoring scale adapted from Hamon et al. [60]. Aerial images were collected on the same day using a quadcopter unmanned aerial system (AgBot, ATI Inc., Oregon City, OR, USA) with a five-band multispectral camera (RedEdge^TM^, Micasense Inc., Seattle, WA, USA). Two features, Multispectral.NDVI and Multispectral.canopy, were extracted from the images [58].

### 4.5. Genotyping

Total DNA were extracted from each lentil accession using the DNeasy 96 Plant Kit (QIAGEN, Valencia, CA, USA) from approximately 0.1 g of young leaf tissue collected from fourteen-day old plants grown in controlled conditions (25 °C 16 h/23 °C 8 h). DNA concentration was quantified using a NanoDrop ND-1000 spectrophotometer (Nano-Drop Technologies, Wilmington, DE, USA) following the manufacturer’s instructions and normalized to 50 ng/µl. GBS libraries were constructed using the two-enzyme approach described in Poland et al. [61] with minor modifications. Five hundred ng of DNA from each line was digested with the two restriction enzymes, *Pst*I and *Msp*I, followed by ligation using 48 barcode adapters with 4–9 bp sequence [61]. Then, 5 µl of ligated DNA from each line was multiplexed in a single tube and then cleaned up using AMPure XP beads (Beckman Coulter, High Wycombe, UK). The multiplexed DNA was amplified using Illumina primers followed by purification. The GBS libraries of the association mapping population were sequenced on an Illumina HiSeq^TM^ 2500 platform using single-read 100 bp at the Washington State University Genomics Core Lab. The GBS libraries of the RIL population were sequenced on an Illumina HiSeq^TM^ 4000 platform using paired-end reads 150 bp long at Novogene Bioinformatics Technology Co., Ltd, Beijing, China. 

Two reference-based GBS pipelines, Stacks and FreeBayes, were used for SNP discovery of the association mapping population and RIL population, respectively. For the Stacks pipeline (the association mapping population), raw sequencing reads with intact barcodes were demultiplexed, cleaned, and truncated to 80 bp with the process_radtags function in Stacks (v 2.0) [62]. Demultiplexed reads were then aligned to the lentil reference genome pre-release v1.2 [20] using the Burrows–Wheeler Aligner [63]. Aligned sequences were filtered using SAMtools (v 0.1.19) [64] to discard multiple mapped reads. SNPs were called using ref_map.pl function with default parameters. For the FreeBayes pipeline (the RIL population), raw reads were demultiplexed and cleaned with process_radtags in Stacks, followed by aligning to the lentil reference genome with Burrows–Wheeler Aligner. SAMtools was used to convert Sequence Alignment Map (SAM) files to Binary Alignment Map (BAM) files, sort the BAM files, and discard the multiple mapped reads. Read groups were added to BAM files using Picard (v 2.18). SNPs were called using Freebayes (v1.2) [65] using the following parameters: —min-base-quality 20, —read-mismatch-limit 2, —min-coverage 10, —no-indels, —genotype-qualities, —ploidy 2, —no-mnps, —no-complex. Using an in-house Perl script, the polymorphic SNPs between two parents were kept, whereas the SNPs with unknown and heterozygous genotypes in one or two of the parents were filtered out. To filter the SNPs for further analysis, VCFtools (v 0.1.16) [66] was used for both GBS pipelines with the following criteria: 1) the association mapping population: MAF > 0.05, missing values < 0.3; 2) the RIL population: MAF > 0.3, missing values < 0.2. For the SNP dataset of the association mapping population, the filtered SNPs were imputed using BEAGLE (v 3.3.2) [67].

### 4.6. Statistical Analysis of Phenotypic Data 

The trait data of the RIL population and the association mapping population were analyzed by ANOVA using ordinal logistic regression (R function polr of MASS package [68]) for categorical variables and general linear model (R function lm of stats package and R function lmer of lme4 package [69]) for numerical variables with genotype as a fixed effect and replication as a random effect by the R 3.5.1 program [70]. The normality of residuals was tested using Skewness, Kurtosis, and Shapiro–Wilk tests (R function describe of psych package; shapiro.test). Frequency distribution histograms of mean values of each trait were drawn by the R function hist. The broad-sense heritability (*H^2^*) was calculated as *H^2^*   =  σ_G_^2^/[σ_G_^2^ + σ_e_^2^/*r*], where σ_G_^2^  =  genotypic variance, σ_e_^2^  =  error variance, and *r*  =  number of replicates. Pearson’s correlation coefficients were calculated between each trait within each population (R function cor), and heatmaps of Pearson’s correlation coefficients were drawn using the R function corrplot.

### 4.7. Linkage Map Construction and QTL Mapping

The genetic linkage map was made using the Kosambi mapping function [71] in the R OneMap package [72] with an LOD value of 6 and a recombination frequency less than 0.3. The recombination counting and ordering algorithm was used for ordering the SNPs. QTL were identified with QTL Cartographer 2.5 software (North Carolina State University, Raleigh, NC, USA) [73] using the composite interval mapping method. Significant QTL were determined by two levels of LOD thresholds: (1) LOD > LOD_threshold_ for nominal QTL, where LOD_threshold_ was calculated for each ARR resistance trait by permutation tests (1000 times) at a *p* value of 0.05; (2) 2.5 < LOD < LOD_threshold_ for suggestive QTL. Mapchart (V 2.2) was used to draw the linkage map and place the QTL.

### 4.8. LD, Population Structure, and GWAS

Pairwise LD in the form of *r^2^* between SNPs was estimated within each chromosome using Haploview (v 4.2) [74]. The LD decay curve was assessed using the method of Hill and Weir [75] in R 3.5.1 program [70] with $\left(r^{2}\right)=\left[\frac{10+C}{\left(2+C\right)\left(11+C\right)}\right]\left[1+\frac{\left(3+C\right)\left(12+12C+C^{2}\right)}{n\left(2+C\right)\left(11+C\right)}\right]$, where *n* is sample size, and *C* indicates the product of the recombination parameter (*4N_e_r*) and the genetic distance. The LD decay rate over each chromosome was measured at *r*^2^ = 0.5. Population structure was estimated using PCA and kinship relatedness matrix generated from MLM in the Genome Association and Prediction Integrated Tool (GAPIT) R package [76]. A three-dimensional PCA plot was drawn using a plot3d function of rgl package in R program. A kinship relatedness matrix was calculated with the VanRaden algorithm in GAPIT. 

GWAS was performed using a multi-locus model, FarmCPU [77], implemented in the R package FarmCPU. SNPs with missing values < 0.3 and MAF > 0.05 were used to perform GWAS in the association mapping population. The first three PCs were used as covariates in this model. The MTAs were assessed by three levels of *p* values: (1) experiment-wise *p* < 2.2 × 10^−6^ (−log10 *p* > 5.7) for major MTAs; (2) marker-wise *p* < 0.0001 (−log10 *p* > 4) for nominal MTAs; (3) marker-wise *p* < 0.001 (−log10 *p* > 3) for suggestive MTAs. The experiment-wise threshold was set according to a Bonferroni-corrected threshold at *p* = 0.01 corresponded to a threshold of −log10 (α/n) > 5.7, with α = 0.01 and n = 4558, the number of markers. The LD blocks were defined using a four gamete rule algorithm [78] in Haploview (v 4.2) [74].

### 4.9. Haplotype Analysis

LD haplotypes were identified among all the accessions of the association mapping population at each LD block using imputed genotypic data. For each haplotype, the phenotypic mean values were calculated for associated traits in a given LD block. The Tukey-HSD test (α = 5%) was used to perform multiple mean comparisons among haplotypes in each LD block. Favorable and unfavorable haplotypes were defined as follows: (1) carrying favorable and unfavorable alleles; (2) representing more than seven lentil accessions (2% of total number of lentil accessions); (3) showing significantly higher or lower values than other haplotypes. Each accession was thereafter described for the number of favorable and unfavorable alleles in each LD block. To classify all the lentil accessions into three groups (resistant-Res, intermediate-Int, susceptible-Sus), a multiple factor analysis (MFA) was performed for the associated traits (RRI, RDL, RGB.blue, RGB.saturation, and RGB.RPL) using the R package FactoMineR [79]. The Res group included the accessions with MFA.dim1 < −2, whereas the Int group and the Sus group referred to −2 < MFA.dim1 < 2 and MFA.dim1 > 2, respectively. A Tukey-HSD test was used to compare the mean numbers of favorable alleles in the three groups. 

### 4.10. Prediction of Candidate Genes and Expression Analysis

The lentil reference genome pre-release v1.2 (http://knowpulse.usask.ca) was used to predict putative genes in each LD block. Putative protein functions were assigned to the SNPs underlying the putative genes. To validate the response of each candidate gene to ARR, expression levels of the genes were explored using qRT-PCR. Briefly, the seeds of two partially resistant accessions (PI 432001 and ILL 5509) and two susceptible accessions (PI 320935 and PI 431824) were grown in moist autoclaved ragdolls in a controlled condition (25 °C 16 h/23 °C 8 h). The roots of seven-day-old lentil seedlings were incubated in 50 mL *A. euteiches* zoospore suspension (10^4^ spores/ ml) for 30 min or in 50 mL autoclaved distilled water for 30 min, followed by harvesting at 6 hpi and 24 hpi. Each treatment was carried out in three replications consisting of 10 lentil roots. The roots were cut about 2 cm from the tips and stored at −80 °C. Total RNA was extracted using RNeasy Plant Mini Kit (Qiagen, Hilden, Germany) followed by DNAse treatment using Turbo DNA-free^TM^ kit (Ambion, Austin, TX, USA). cDNA was synthesized from total RNA of each sample using the High-Capacity cDNA Reverse Transcription Kit with RNase Inhibitor (Applied Biosystems, Foster City, CA, USA). qRT-PCR was performed in 10 µl reaction volumes with 2 µl of diluted cDNA (20x dilution for *LRR-RLK*, *ABCA*, and *CHI*; 4x dilution for *CYP71* and *PE*), 500 nM of each primer, and 5 µl Ssofast^TM^ EvaGreen supermix (Bio-Rad Laboratories Inc., Hercules, CA, USA). The qRT-PCR reactions were conducted on Bio-Rad CFX96 Real-Time PCR System (Hercules, CA, USA) using the following conditions: 95 °C for 1 min, 40 cycles of 95 °C for 5 s, and 60 °C for 20 s. Melting curve analysis was performed by increasing the temperature from 65 °C to 95 °C in increments of 0.5 °C for 5 s. qRT-PCR primers were designed for targeted lentil genes to amplify amplicons using Primer 3 (v 0.4.0) [80] (Supplementary Table S7). Each qRT-PCR reaction was replicated two times for each biological replicate in each treatment. Relative expression values for target genes were calculated using the $2^{-∆∆CT}$ method [81], and the expression of ß-tubulin-3 was used for normalization [82].

## 5. Conclusions

Taken together, this two-pronged approach based on linkage analysis and association mapping enabled us to dissect comprehensively the genetic architecture of ARR resistance in lentil. In this study, we identified 19 QTL-mapping-based QTL and 38 GWAS-based QTL using both traditional phenotyping traits and image-derived features. Seven QTL clusters were discovered on six chromosomes. Gene expression analysis used to explore five putative candidate genes indicated three of them (*ABCA*, *CHI,* and *PE*) were differentially expressed at early stages of infection, and two of them (*ABCA* and *PE*) may be associated with ARR resistance. This study provides valuable genomic resources to aid the development of lentil varieties resistant to ARR. Future functional analyses of the candidate genes will enable us to illustrate more completely the molecular mechanisms in lentil for ARR resistance.

## Figures and Tables

**Table 1:** Statistical analysis results of all the Aphanomyces root rot (ARR) resistance traits in the recombinant inbred line (RIL) population and the association mapping population.

Population	Trait ^a^	Number of Lines	Number of Observations	Min ^b^	Max ^b^	Mean ^b^	SE ^b^	Skew ^c^	Kurtosis ^c^	Normality Test ^d,f^	G Effect ^e,f^	R Effect ^e,f^	H^2^ ^e^
RIL	RRI	189	1564	0	5	1.54	0.04	0.19	0.30	ns	***	ns	0.24
	SDL	189	551	1	4	1.69	0.03	0.76	0.70	***	***	*	0.10
	RDL	189	554	1	4	1.9	0.04	0.71	1.02	***	***	*	0.13
	RGB.blue	189	1563	131.19	197.79	171.2	0.27	−0.21	−0.18	ns	***	***	0.52
	RGB.saturation	189	1563	0.05	0.17	0.10	0.00	0.30	−0.24	ns	***	ns	0.47
	RGB.SPL	189	541	1	5	1.54	0.03	0.58	−0.13	***	***	ns	0.17
	RGB.RPL	189	541	1	5	1.65	0.03	0.72	−0.02	***	***	ns	0.21
	AGI	173	505	0.31	4.81	2.58	0.03	0.09	−0.20	ns	***	*	0.13
	Multispectral.NDVI	173	497	0.01	0.24	0.12	0.00	0.06	−0.11	ns	**	ns	0.13
	Multispectral.canopy	173	497	−0.19	0.93	0.33	0.01	−0.04	0.25	ns	*	*	0.05
Association	RRI	326	3052	0	5	3.35	0.01	−1.05	2.07	***	***	***	0.73
	SDL	326	2910	1	5	2.19	0.02	0.28	−0.81	ns	***	***	0.53
	RDL	326	2911	1	5	2.11	0.02	0.62	−0.62	***	***	***	0.50
	RGB.blue	326	3052	50.53	119.67	79.82	0.20	0.41	−0.06	***	***	***	0.62
	RGB.saturation	326	3052	0.31	0.43	0.38	0.00	−0.20	0.12	**	***	***	0.73
	RGB.SPL	326	2895	1	5	2.57	0.02	−0.08	−0.78	*	***	***	0.50
	RGB.RPL	326	2895	1	5	2.52	0.02	0.16	−0.93	ns	***	***	0.54

^a^ ARR resistance traits are abbreviated as follows: RRI-root rot index, SDL-shoot dry weight loss per plant, RDL-root dry weight loss per plant, RGB.blue-average intensity of blue channel acquired from an RGB camera, RGB.saturation-standard deviation of saturation channel acquired from an RGB camera, RGB.SPL-number of pixels loss per plant in shoot acquired from an RGB camera, RGB.RPL-number of pixels loss per plant in root acquired from an RGB camera, AGI-above ground index, Multispectral.canopy-canopy area acquired from a multispectral camera, Multispectral.NDVI-standard deviation of normalized difference vegetation index acquired from a multispectral camera. ^b^ Minimum, maximum, mean, and standard error. ^c^ Skew and Kurtosis values used for testing normality. ^d^ Normality tested by Shapiro–Wilk test. ^e^ G effect: genotype effect; R effect: replicate effect; H^2^: heritability. ^f^ *** < 0.001, ** < 0.01, * < 0.05, not significant (ns) > 0.05.

**Table 2:** Quantitative trait loci (QTL) for ARR resistance traits in the RIL population across two environments.

Trait	QTL ^a^	Environment ^b^	Closest Marker	Chr	Position	LOD ^c^	R^2^	CI ^d^	Parental Allele ^e^
RRI	Q.RRI-Lc2.1	CC	LcChr2-10483056	2	10483056	3.2**	6.2%	22.8−31.0	K191-2
	Q.RRI -Lc5.1	CC	LcChr5-229370222	5	229370222	2.7*	5.3%	62.0−64.8	K192-1
SDL	Q.SDL-Lc3.1	CC	LcChr3-124302109	3	124302109	2.8*	5.9%	81.8−83.9	K192-1
RGB.blue	Q.BLU-Lc2.1	CC	LcChr2-10483056	2	10483056	3.3**	6.5%	22.8−30.8	K192-1
	Q.BLU-Lc5.1	CC	LcChr5-257437930	5	257437930	3.5**	6.9%	110.6−119.7	K192-1
	Q.BLU-Lc7.1	CC	LcChr7-93158569	7	93158569	3.1**	6.0%	48.8−60.0	K192-1
RGB.saturation	Q.SAT-Lc2.1	CC	LcChr2-8058084	2	8058084	2.7*	5.2%	22.8−23.8	K191-2
	Q.SAT-Lc3.1	CC	LcChr3-65935857	3	65935857	2.7*	5.7%	35.0−38.1	K192-1
	Q.SAT-Lc7.1	CC	LcChr7-93158569	7	93158569	4.2**	8.1%	47.0−61.0	K191-2
RGB.SPL	Q.SPL-Lc2.1	CC	LcChr2-4851535	2	4851535	2.6*	5.3%	13.1−13.1	K191-2
RGB.RPL	Q.RPL-Lc4.1	CC	LcChr4-83603021	4	83603021	4.8**	9.5%	35.5−53.2	K192-1
	Q.RPL-Lc4.2	CC	LcChr4-175357959	4	175357959	2.8*	5.7%	71.44−71.44	K191-2
AGI	Q.AGI-Lc2.1	Field	LcChr2-10483056	2	10483056	2.7*	5.6%	28.7−28.8	K191-2
	Q.AGI-Lc5.1	Field	LcChr5-229370222	5	229370222	3.4**	7.0%	51.7−63.1	K192-1
Multispectral.canopy	Q.CAN-Lc2.1	Field	LcChr2-10483056	2	10483056	2.6*	5.7%	29.8−31.0	K192-1
	Q.CAN-Lc7.1	Field	LcChr7-61352757	7	61352757	3.2**	6.8%	8.3−16.7	K192-1
Multispectral.NDVI	Q.NDVI-Lc6.1	Field	LcChr6-170967409	6	170967409	4.1**	8.8%	77.0−86.8	K191-2
	Q.NDVI-Lc6.2	Field	LcChr6-196641316	6	196641316	5.6**	12.1%	104.1−120.0	K191-2
	Q.NDVI-Lc7.1	Field	LcChr7-63933214	7	63933214	3.7**	7.8%	22.2−33.6	K192-1

^a^ QTL names are assigned by the analysis method (QTL-mapping), ARR related trait, the initial of genus and species (*Lens culinaris*), chromosome numbers, and the order of the QTL. ^b^ CC represents controlled conditions. ^c^ ** represents nominal QTL, where LOD > LOD_threshold_. The LOD_threshold_ values are 3.06 (RRI), 3.11 (SDL), 3.18 (RGB.blue), 3.07 (RGB.saturation), 3.14 (RGB.SPL), 3.00 (RGB.RPL), 3.07 (AGI), 3.01 (Multispectral.canopy), and 2.98 (Multispectral.NDVI) * indicates suggestive QTL, where 2.5 < LOD < LOD_threshold_. ^d^ CI is the confidence interval of QTL regions. ^e^ Parental allele contributing to the trait.

**Table 3:** QTL for the ARR resistance traits in the association mapping population.

Trait	QTL^a^	Trait-Associated Marker	Chr	Position	CI ^b^	Number of Markers ^c^	*p*-Value ^d^	MAF ^e^	R^2^	Favorable Allele ^f^
RRI	G.RRI-Lc.1.1	1569_6	1	72094185	71880185-72308185	2	3.4 × 10^−10^ ***	24%	10.7%	G/**A**
	G.RRI-Lc.1.2	869_19	1	271625013	271384013-271839013	3	6 × 10^−5^ **	18%	3.9%	G/**A**
	G.RRI-Lc.2.1	1827_75	2	102752889	102361889-103143889	7	1.4 × 10^−5^ **	39%	19.3%	T/**C**
	G.RRI-Lc.2.2	2799_53	2	283548685	283157685-283939685	2	1.7 × 10^−4^ *	27%	13.4%	G/**T**
	G.RRI-Lc.4.1	5009_13	4	109679991	109524991-109834991	6	2.6 × 10^−4^ *	25%	1.4%	**G**/A
	G.RRI-Lc.5.1	7154_47	5	225981924	225823924-226139924	2	2.7 × 10^−5^ **	20%	10.0%	T/**A**
	G.RRI-Lc.6.1	9084_41	6	51508203	50746203-52270203	4	3.6 × 10^−4^ *	5%	3.1%	C/**T**
	G.RRI-Lc.6.2	8442_65	6	155091517	154329517-155853517	4	9.2 × 10^−10^ ***	49%	2.3%	A/**C**
	G.RRI-Lc.7.1	9286_7	7	101144016	101016016-101272016	1	3.8 × 10^−4^ *	6%	8.4%	A/**G**
RDL	G.RDL-Lc.1.1	1604_28	1	75421049	75207049-75635049	2	4.6 × 10^−5^ **	11%	5.5%	G/**T**
	G.RDL-Lc.2.1	2065_7	2	14688732	14369732-15007732	4	1.3 × 10^−4^ *	6%	6.5%	G/**A**
	G.RDL-Lc.2.2	2066_11	2	146997456	146678456-147316456	1	1.6 × 10^−4^ *	17%	4.9%	C/**T**
	G.RDL-Lc.2.3	2770_18	2	279028234	278709234-279347234	1	8.4 × 10^−7^ ***	24%	2.4%	C/**T**
	G.RDL-Lc.3.1	4445_35	3	192079718	191596718-192562718	5	4.1 × 10^−4^ *	14%	2.3%	G/**T**
	G.RDL-Lc.4.1	5190_31	4	145662855	145507855-145817855	1	2.1 × 10^−4^ *	37%	3.3%	**A**/T
	G.RDL-Lc.6.1	9009_9	6	40654874	39892874-41416874	3	5 × 10^−6^ **	45%	4.2%	A/**G**
	G.RDL-Lc.7.1	10617_29	7	9677731	9549731-9805731	1	2.3 × 10^−4^ *	31%	6.4%	**C**/T
RGB.saturation	G.SAT-Lc1.1	1323_60	1	329974767	329760767-330188767	4	5.1 × 10^−5^ **	29%	4.2%	G/**A**
	G.SAT-Lc2.1	1913_55	2	1176134	857134-1495134	7	5 × 10^−5^ **	48%	8.3%	**G**/A
	G.SAT-Lc2.2	3450_21	2	9515161	9196161-9834161	8	2.1 × 10^−4^ *	17%	21.0%	**A**/T
	G.SAT-Lc4.1	5069_30	4	123435206	123280206-123590206	2	2 × 10^−7^ ***	36%	1.7%	**T**/C
	G.SAT-Lc5.1	7154_47	5	225981924	225823924-226139924	2	8.4 × 10^−9^ ***	20%	3.5%	T/**A**
	G.SAT-Lc5.2	7541_42	5	258022703	257864703-258180703	5	2.3 × 10^−6^ **	13%	1.7%	T/**C**
	G.SAT-Lc6.1	9009_9	6	40654874	39892874-41416874	3	2.6 × 10^−6^ **	45%	1.9%	A/**G**
	G.SAT-Lc7.1	10394_17	7	62649254	62521254-62777254	3	3.3 × 10^−5^ **	25%	3.8%	**C**/T
	G.SAT-Lc7.2	9492_47	7	157790680	157662680-157918680	3	5.6 × 10^−7^ ***	39%	21.4%	**A**/T
RGB.blue	G.BLU-Lc1.1	1569_6	1	72094185	71880185-72308185	2	1.1 × 10^−6^ ***	24%	13.7%	G/**A**
	G.BLU-Lc2.1	3136_31	2	41405986	41086986-41724986	1	3.2 × 10^−4^ *	39%	3.2%	**T**/C
	G.BLU-Lc3.1	4814_43	3	75993537	75510537-76476537	3	2.4 × 10^−6^ **	40%	5.9%	T/**A**
	G.BLU-Lc3.2	4403_39	3	189225788	188742788-189708788	4	6.1 × 10^−7^ ***	27%	2.1%	A/**C**
	G.BLU-Lc7.1	9616_9	7	197564498	197436498-197692498	4	1.6 × 10^−4^ *	47%	2.6%	**G**/A
	G.BLU-Lc7.2	10127_27	7	245915689	245787689-246043689	2	7.9 × 10^−5^ **	22%	8.6%	**G**/T
RGB.RPL	G.RPL-Lc2.1	3028_63	2	309553121	309234121-309872121	6	2.1 × 10^−6^ ***	13%	7.1%	C/**T**
	G.RPL-Lc3.1	3562_32	3	110087057	109604057-110570057	2	4.8 × 10^−4^ *	12%	7.7%	C/**T**
	G.RPL-Lc4.1	6453_18	4	9587251	9432251-9742251	2	1.9 × 10^−5^ **	25%	16.2%	T/**C**
	G.RPL-Lc4.2	5190_31	4	145662855	145507855-145817855	1	8.9 × 10^−4^ *	37%	1.9%	**A**/T
	G.RPL-Lc5.1	6649_58	5	127185901	127027901-127343901	1	1.5 × 10^−4^ *	20%	6.2%	**T**/C
	G.RPL-Lc6.1	9009_9	6	40654874	39892874-41416874	3	1.3 × 10^−4^ *	45%	7.8%	A/**G**

^a^ QTL names are assigned by the analysis method (GWAS), ARR related traits, the initial of genus and species (*Lens culinaris*), chromosome number and the order of the QTL. ^b^ CI is the confidence interval of QTL regions derived from ± linkage disequilibrium (LD) decays for each chromosome. ^c^ Number of markers within the LD block. ^d^ ***represents major QTL (*p* < 2.2 × 10^−6^), ** for nominal QTL (*p* < 0.0001), * for suggestive QTL (*p* < 0.001). e Minor allele frequency (MAF). ^f^ Favorable alleles in bold and underlined text.

## Supplementary Materials

Supplementary materials can be found at https://www.mdpi.com/1422-0067/21/6/2129/s1. Figure S1. Frequency distribution of ARR resistance traits in the RIL population and the association mapping population; Figure S2. Linkage disequilibrium decay in the association mapping population; Table S1. Summary of raw sequencing data for the RIL population and the association mapping population; Table S2. Distribution of SNPs on a linkage map for the RIL population; Table S3. Description of LD block haplotypes in the association mapping population; Table S4. LD block haplotype distribution of the association mapping population; Table S5. Putative annotation of SNPs in detected LD blocks; Table S6. Description of the association population; Table S7. Primers used for qRT-PCR in this study.

## Abbreviations

ARR	Aphanomyces root rot
LD	Linkage disequilibrium
GBS	Genotyping by sequencing
SNP	Single nucleotide polymorphism
QTL	Quantitative trait loci
LSP	Lentil single plant-derived
ICARDA	International Center for Agricultural Research in the Dry Areas
RIL	Recombinant inbred line
RGB	Red-Green-Blue
RRI	Root rot index
AGI	Above ground index
SDL	Shoot dry weight loss per plant
RDL	Root dry weight loss per plant
RGB.SPL	Number of pixels loss per plant in shoot
RGB.RPL	Number of pixels loss per plant in root
RGB.blue	Average intensity of blue channel
RGB.saturation	Standard deviation of saturation channel
Multispectral.NDVI	Standard deviation of normalized difference vegetation index
Multispectral.canopy	Canopy area
MAF	Minor allele frequency
ANOVA	Analysis of variance
PCA	Principle component analysis
PCs	Principle components
GAPIT	Genome Association and Prediction Integrated Tool
MTAs	Marker-trait associations
MFA	Multiple factor analysis
qRT-PCR	Quantitative reverse transcriptase-polymerase chain reaction
hpi	Hours post inoculation
dpi	Days post inoculation
LRR-RLK	Leucine Rich Repeat Receptor-like Kinase
CYP71	Cytochrome P450 family 71 protein
ABCA	ABC transporter A family protein
PE	Pectin esterase
CHI	Chalcone-flavanone isomerase family protein
SAM	Sequence Alignment Map
BAM	Binary Alignment Map
