# Supplementary material for: Dissecting the Genetic Architecture of Aphanomyces Root Rot Resistance in Lentil by QTL Mapping and Genome-Wide Association Study
Source: Int J Mol Sci. 2020 Mar 20;21(6):2129. doi: 10.3390/ijms21062129 (PMC7139309; doi:10.3390/ijms21062129)
Supplement: Supplementary file 1 [file ijms-21-02129-s001.zip › Supplementary Figures S1-S2 Table S1 S2 and S7-IJMS.docx]

**Supplementary Figure S1. Frequency distribution of ARR resistance traits in the RIL population and the association mapping population**

Acronyms used for the ARR resistance traits in this study:

RRI, root rot index

AGI, above ground index

SDL, shoot dry weight loss per plant

RDL, root dry weight loss per plant

RGB.blue, average intensity of blue channel acquired from a RGB camera

RGB.saturation, standard deviation of saturation channel acquired from a RGB camera

RGB.SPL, number of pixels loss per plant in shoot acquired from a RGB camera

RGB.RPL, number of pixels loss per plant in root acquired from a RGB camera

Multispectral.NDVI, standard deviation of normalized difference vegetation index acquired from a multispectral camera

Multispectral.canopy, canopy area acquired from a multispectral camera

1. The RIL population


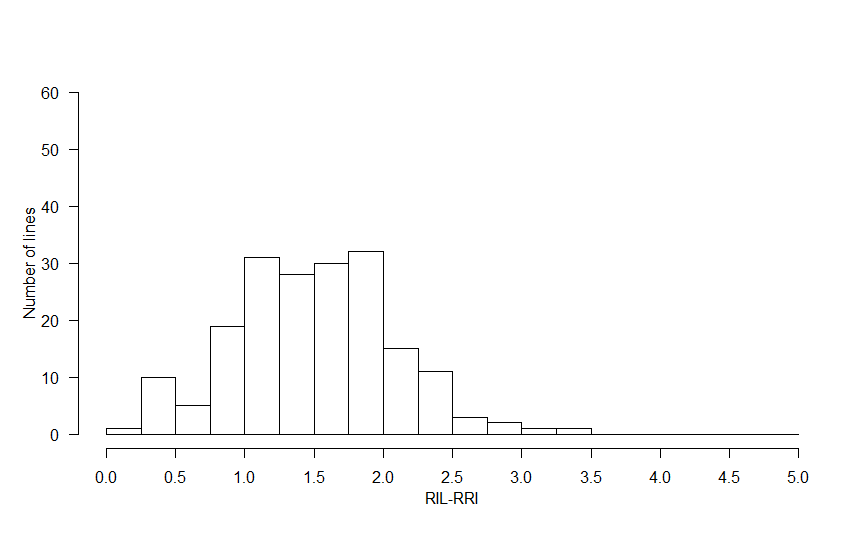

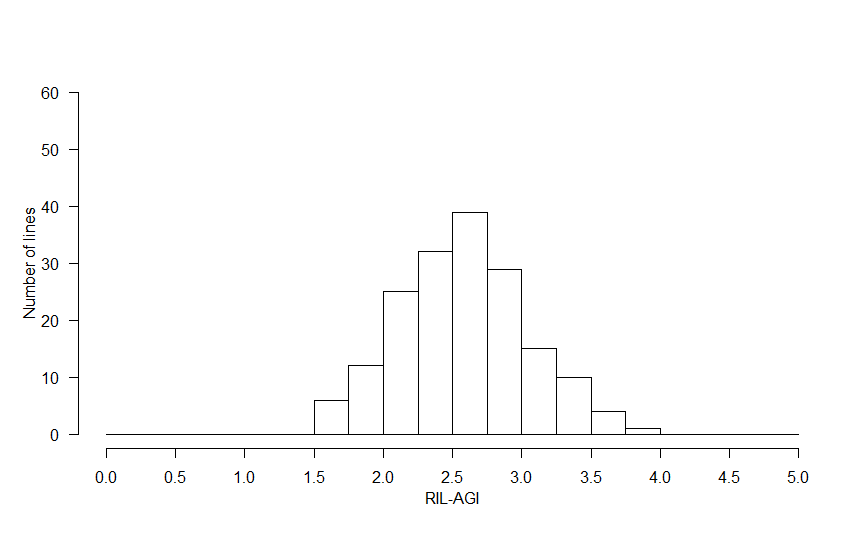

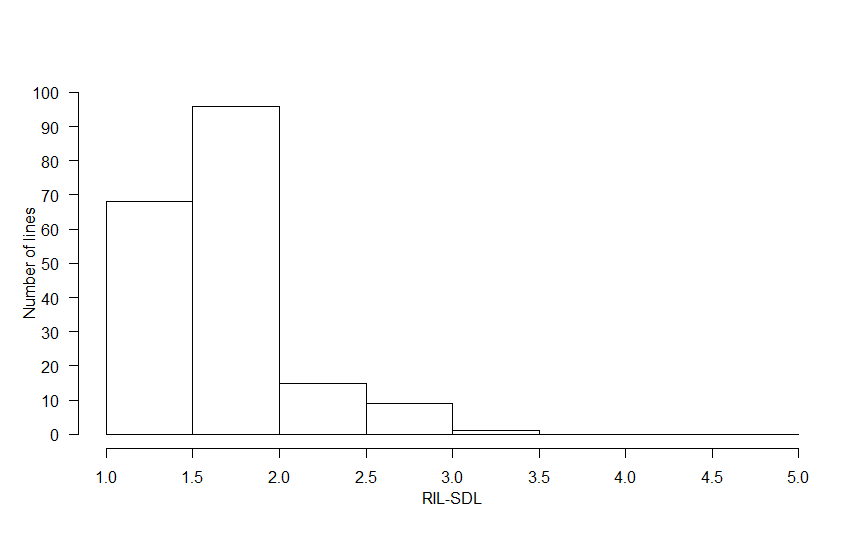

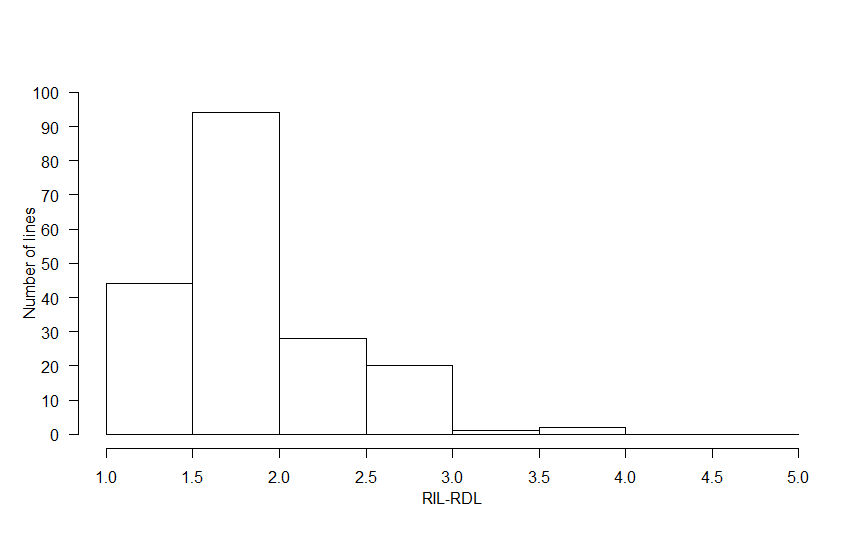

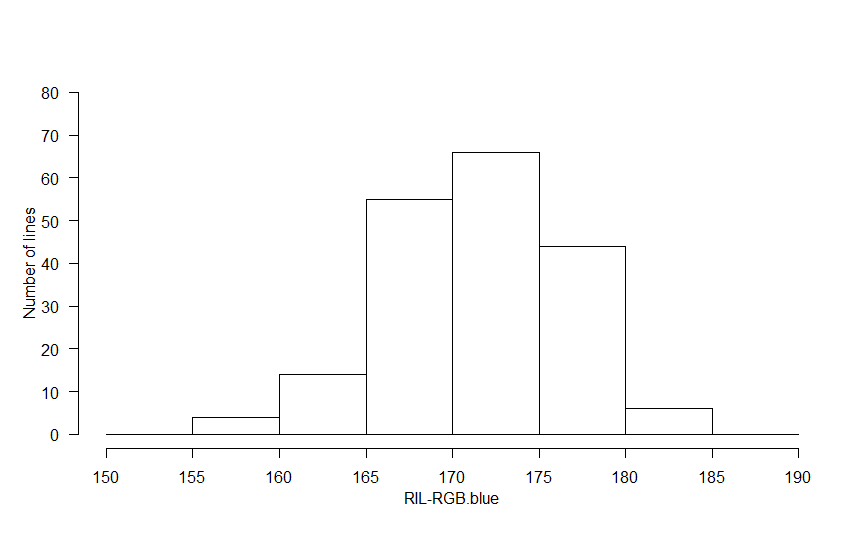

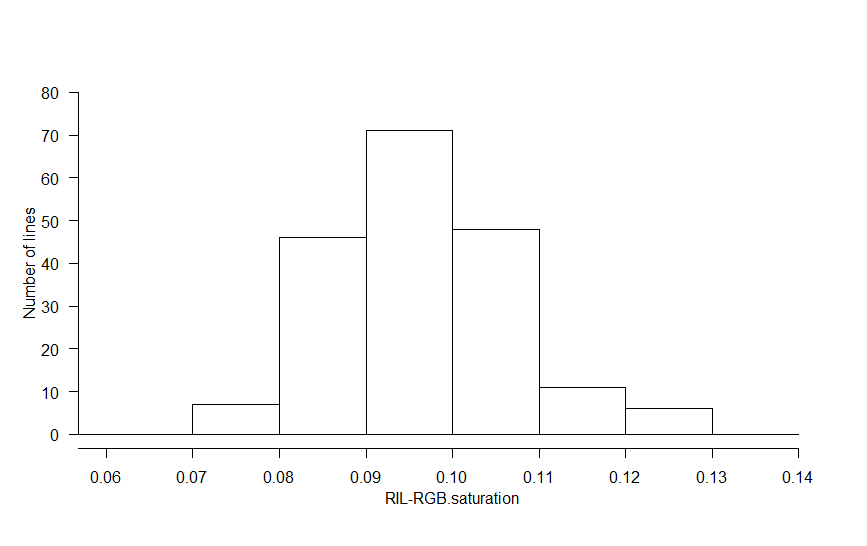


K192-1

K192-1

K192-1

K191-2

K192-1

K192-1

K192-1

K192-1

K191-2

K191-2

K191-2

K191-2

K191-2


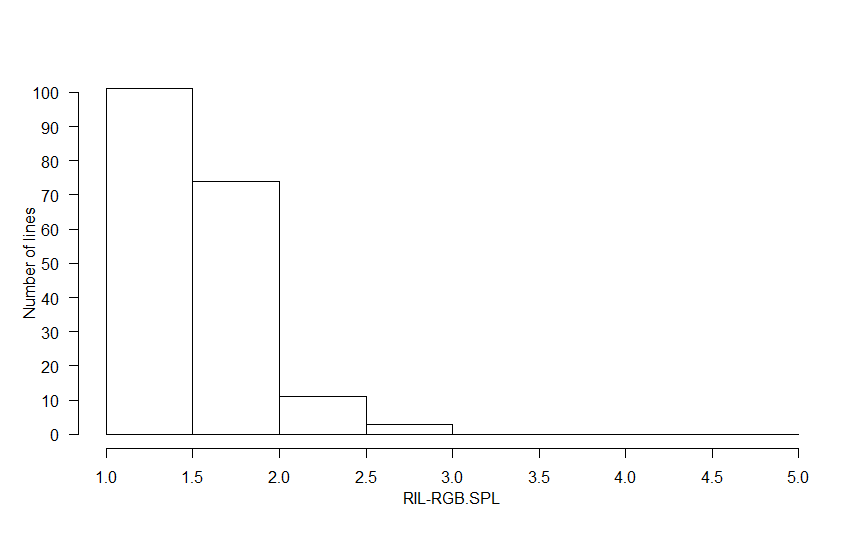

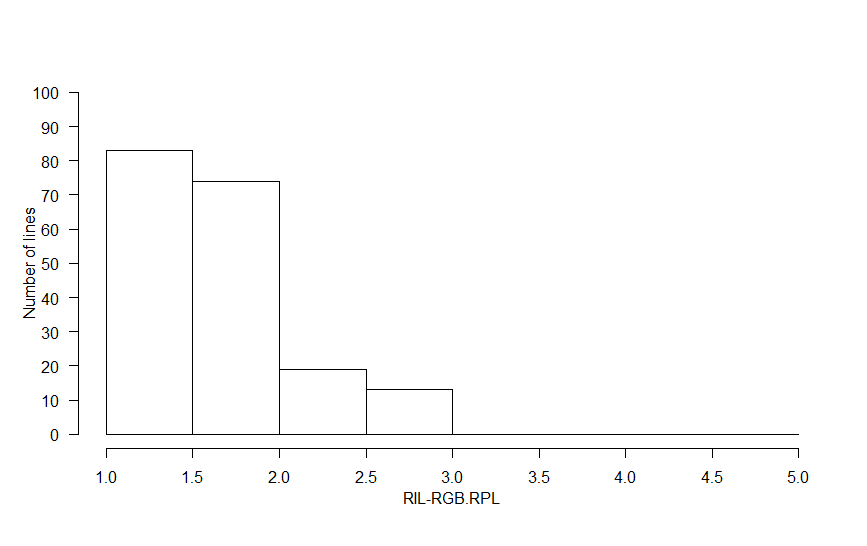


K191-2

K192-1

K191-2


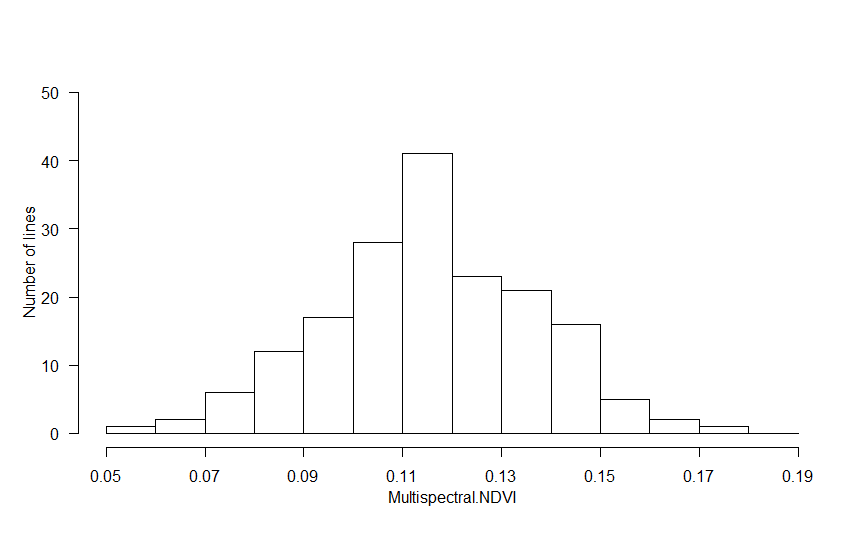

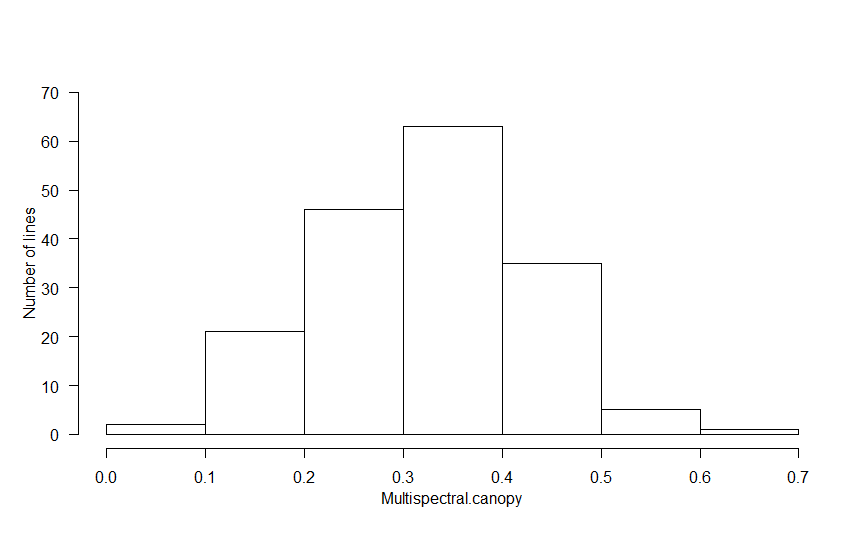


K191-2

K191-2

K192-1

K192-1

1. The association mapping population


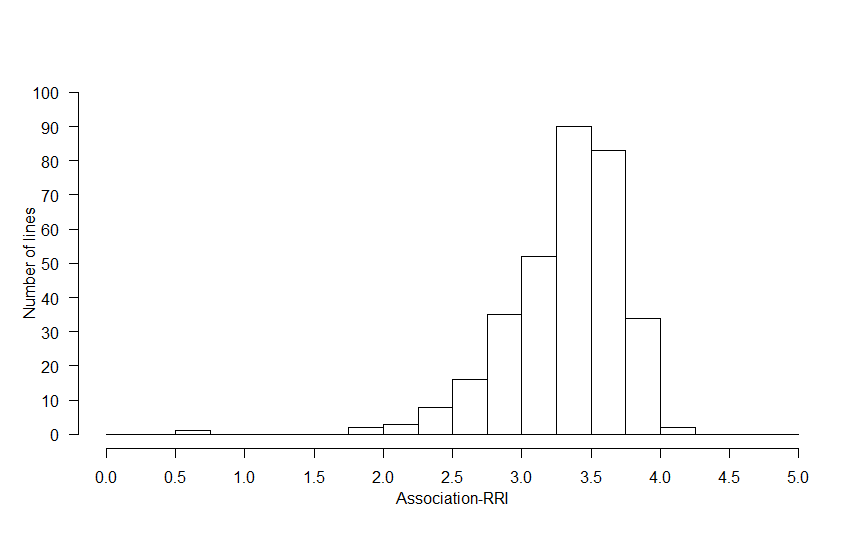

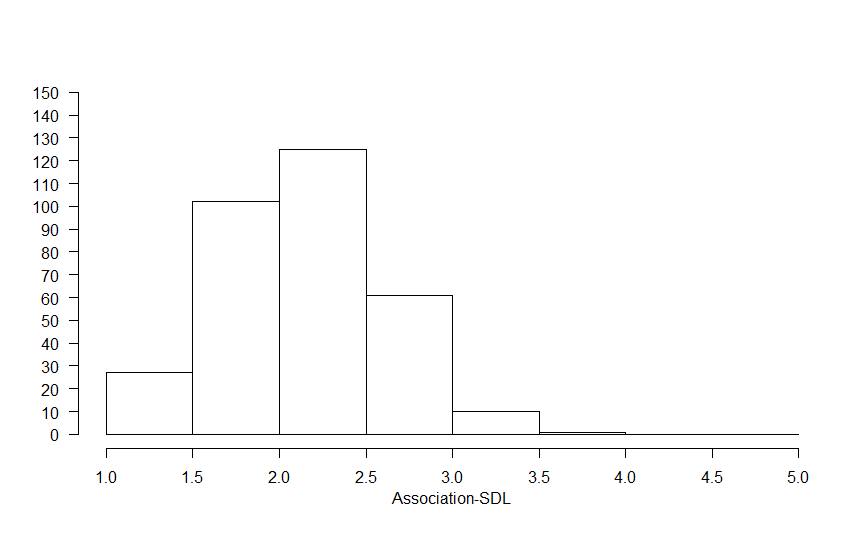

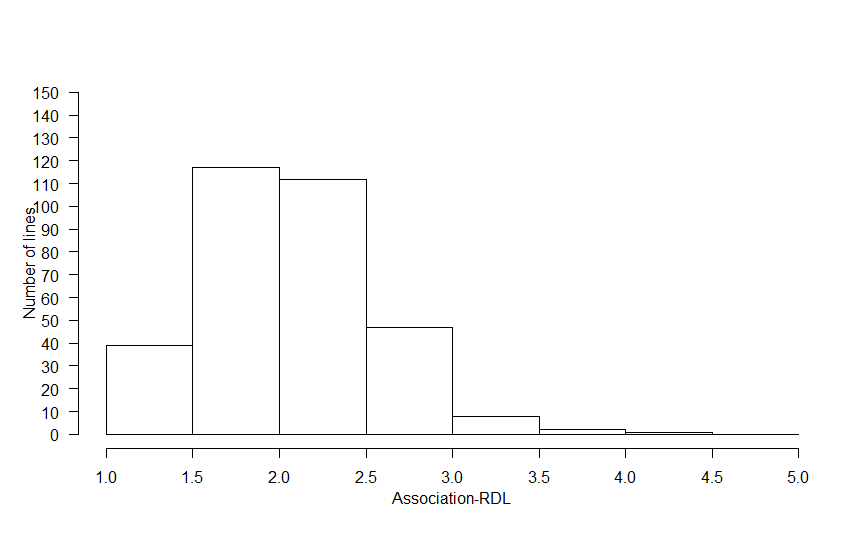

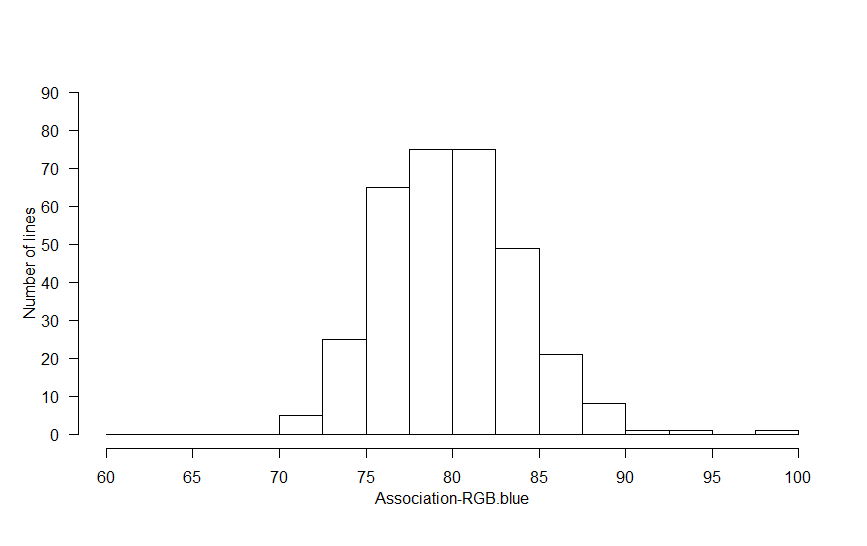

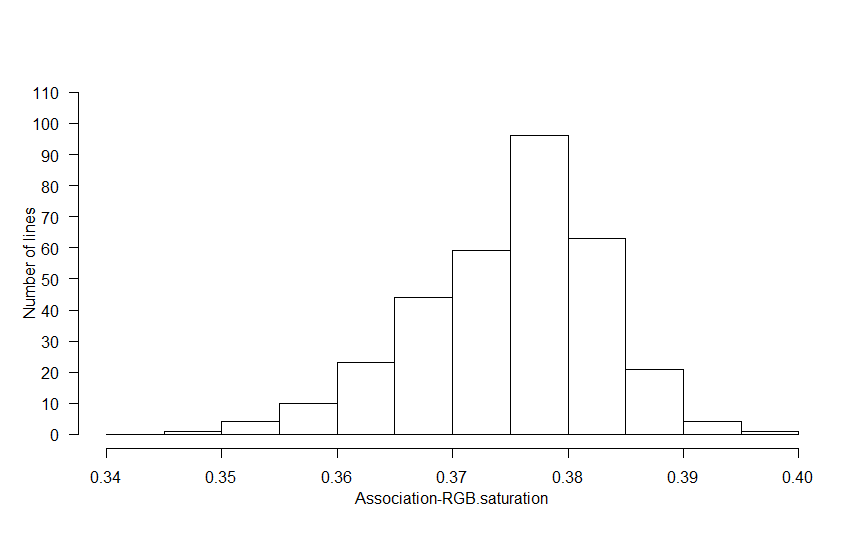

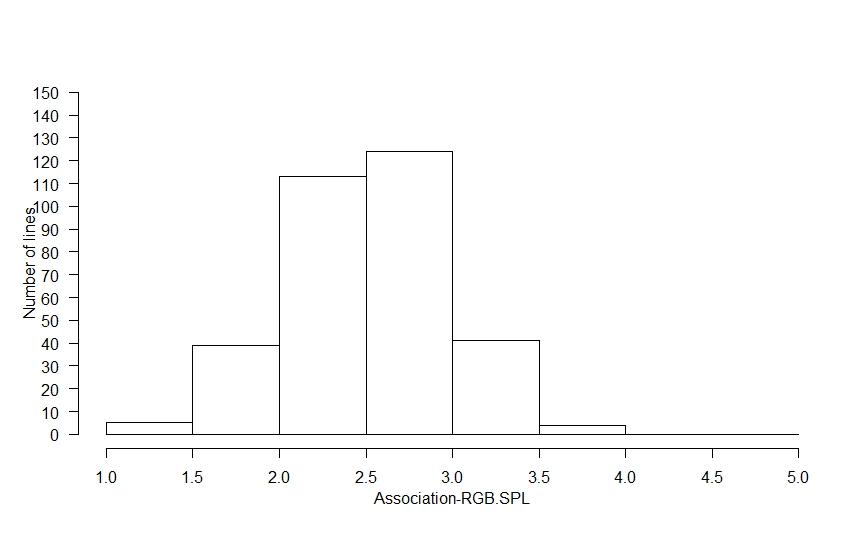


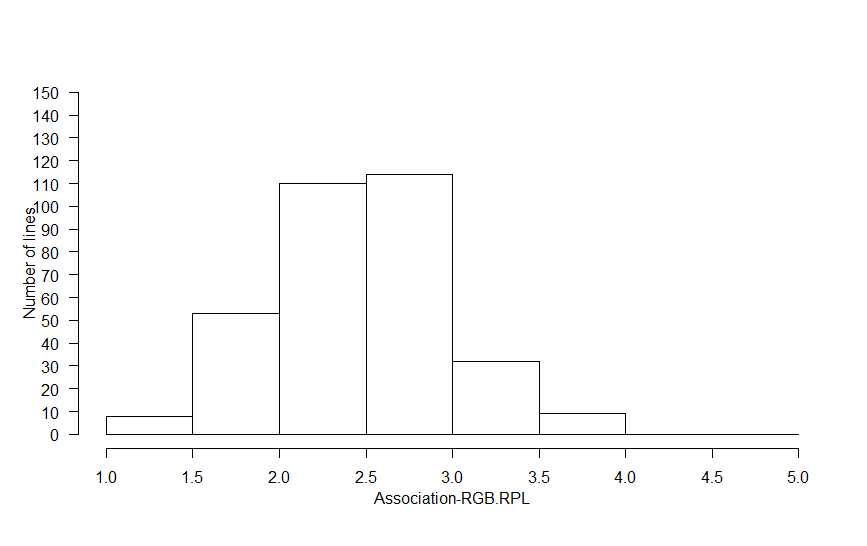


**Supplementary Figure S2. Linkage disequilibrium decay in the association mapping population.** Colored curves indicate the estimated LD decay for each chromosome. Dashed red lines represent LD decay rate measured at *r*^2^ = 0.5.


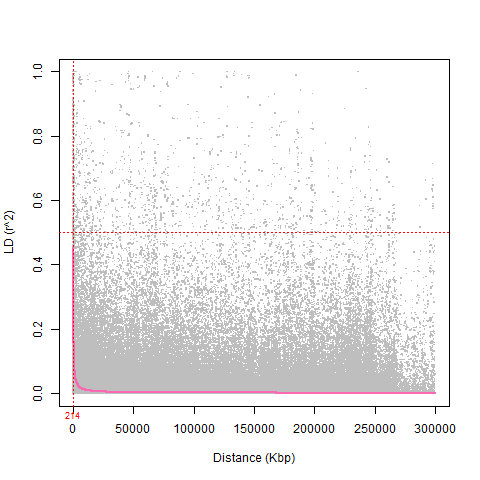

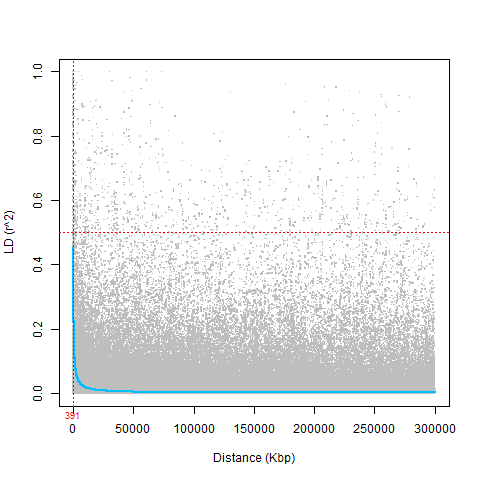

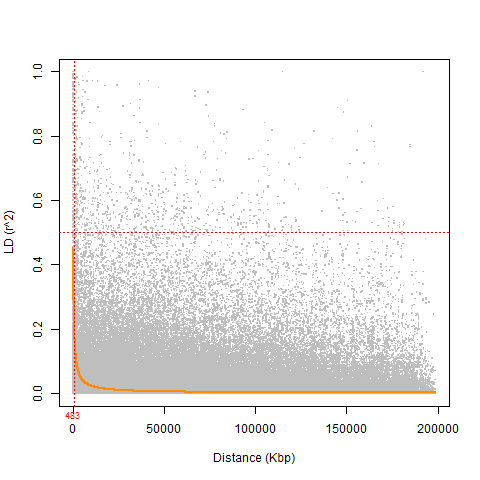

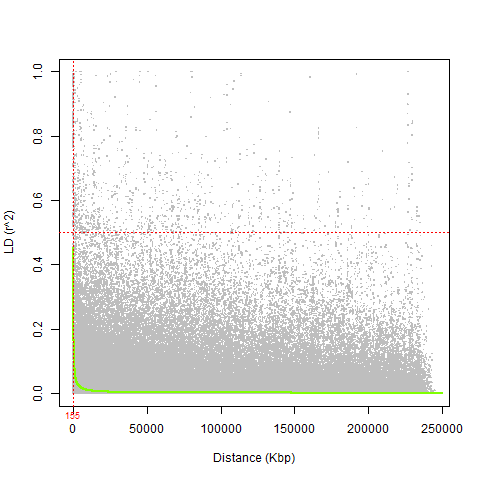

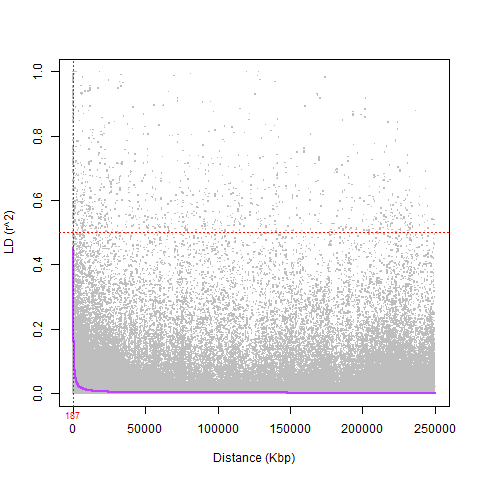

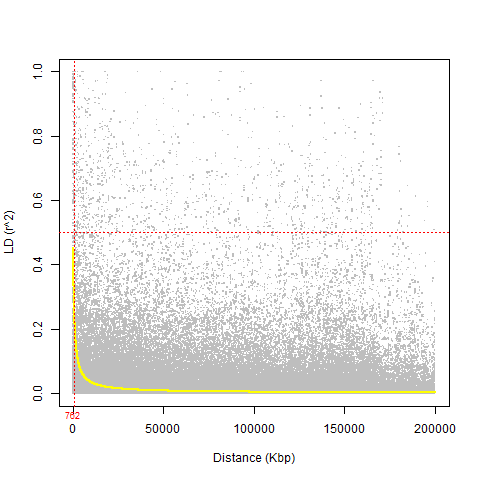

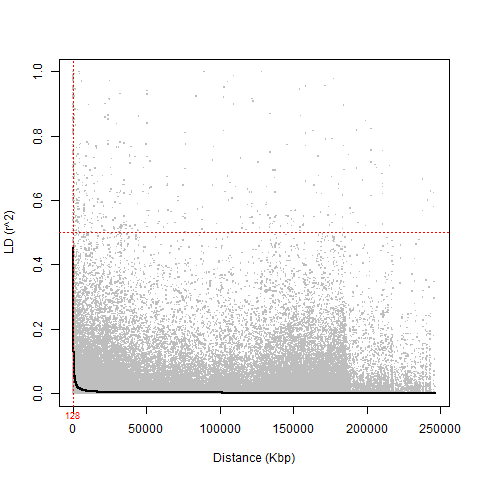


Chr 4

Chr1 1

Chr 3

Chr 2

Chr 6

Chr 7

Chr 5

**Supplementary Table S1. Summary of raw sequencing data for the RIL population and the association mapping population**

| GBS library | Raw Reads | Error Rate (%) | Q20 (%) | Q30 (%) | GC Content (%) |
| --- | --- | --- | --- | --- | --- |
| RIL-1 | 984117230 | 1.97 | 96.63 | 93.02 | 43.36 |
| RIL-2 | 990793106 | 1.91 | 96.67 | 93.17 | 43.59 |
| RIL-3 | 987151360 | 2.00 | 96.46 | 92.74 | 43.56 |
| RIL-4 | 989544608 | 3.10 | 96.42 | 92.74 | 43.68 |
| Association-1 | 337973016 | 0.23 | 98.35 | 96.50 | 43.98 |
| Association-2 | 261602192 | 0.08 | 96.52 | 92.85 | 43.59 |
| Association-3 | 240748898 | 1.98 | 95.99 | 91.82 | 41.31 |
| Association-4 | 204190649 | 0.08 | 96.12 | 92.10 | 42.33 |
| Association-5 | 191186024 | 0.16 | 97.76 | 95.30 | 42.86 |
| Association-6 | 234244625 | 0.53 | 84.65 | 76.22 | 41.23 |
| Association-7 | 215292545 | 0.90 | 85.65 | 77.66 | 41.54 |

**Supplementary Table S2. Distribution of SNPs on a linkage map for the RIL population**

|  | LGI-chr1 | LGII-chr2 | LGIII-chr3 | LGIV-chr4 | LGV-chr5 | LGVI-chr6 | LGVII-chr7 | Total |
| --- | --- | --- | --- | --- | --- | --- | --- | --- |
| Number of markers | 314 | 443 | 502 | 534 | 401 | 323 | 348 | 2865 |
| Length (cM) | 145.2 | 145.2 | 159.8 | 151.7 | 125.6 | 139.1 | 111.5 | 978.1 |
| Number of marker per cM | 2.2 | 3.1 | 3.1 | 3.5 | 3.2 | 2.3 | 3.1 | 2.9 |
| Number of gaps (>10 cM) | 0 | 0 | 0 | 1 | 0 | 1 | 0 | 2 |
| Number of gaps (>5 cM) | 0 | 2 | 4 | 3 | 2 | 3 | 2 | 16 |

**Supplementary Table S7. Primers used for qRT-PCR in this study**

| **Description** | **Forward primer** | **Reverse primer** | **Amplicon (bp)** |
| --- | --- | --- | --- |
| *ABC transporter A family protein (ABCA)* | CGGGTTTACACCATGCTCTT | GATGCCAAGCTAGCCATGAT | 119 |
| *Cytochrome P450 family 71 protein (CYP71)* | AAAACGTGTCCAATCGTTCC | TCGTGTTCGTTCCTTTTTCC | 165 |
| *LRR receptor-like kinase (LRR-RLK)* | TCAACTAACGGGCGGTATTC | GTTCAAGCTGGAAGGCACTC | 106 |
| *Chalcone-flavanone isomerase family protein (CHI)* | TTGGAAGCTGCAGTGTTGAC | TTCATCAAGCCCCAAATCTC | 176 |
| *Pectin esterase (PE)* | CTCCAAAGCATGCAAATCAA | CTCGGCGTGGTTTAGAGAAG | 199 |
